# Reed & Carnrick’s Preparation

**Published:** 1889-07

**Authors:** 


REED & CARNRICK'S LACTO-PREPARATION.—See the advertisement of this new preparation put forth by the above excellent House. It does not replace their deservedly popular preparation, "*Soluble Food*," but is especially adapted for children under eight months of age.

